# Automated tuning of an eight-channel cardiac transceive array at 7 tesla using piezoelectric actuators

**DOI:** 10.1002/mrm.25356

**Published:** 2014-07-01

**Authors:** Graeme A Keith, Christopher T Rodgers, Aaron T Hess, Carl J Snyder, J Thomas Vaughan, Matthew D Robson

**Affiliations:** 1 Oxford Centre for Clinical Magnetic Resonance Research, University of OxfordJohn Radcliffe Hospital, Oxford, United Kingdom; 2 Center for Magnetic Resonance Research, University of MinnesotaMinneapolis, Minnesota, USA.

**Keywords:** parallel transmit, ultra-high field, 7 Tesla, tuning, RF coil, piezo-actuator

## Abstract

**Purpose:**

Ultra-high field (UHF) MR scanning in the body requires novel coil designs due to B_1_ field inhomogeneities. In the transverse electromagnetic field (TEM) design, maximum B_1_ transmit power can only be achieved if each individual transmit element is tuned and matched for different coil loads, which requires a considerable amount of valuable scanner time.

**Methods:**

An integrated system for autotuning a multichannel parallel transmit (pTx) cardiac TEM array was devised, using piezoelectric actuators, power monitoring equipment and control software. The reproducibility and performance of the system were tested and the power responses of the coil elements were profiled. An automated optimization method was devised and evaluated.

**Results:**

The time required to tune an eight-element pTx cardiac RF array was reduced from a mean of 30 min to less than 10 min with the use of this system.

**Conclusion:**

Piezoelectric actuators are an attractive means of tuning RF coil arrays to yield more efficient B_1_ transmission into the subject. An automated mechanism for tuning these elements provides a practical solution for cardiac imaging at UHF, bringing this technology closer to clinical use. Magn Reson Med 73:2390–2397, 2015. © 2014 The Authors. Magnetic Resonance in Medicine published by Wiley Periodicals, Inc. on behalf of International Society for Magnetic Resonance in Medicine.

## INTRODUCTION

Cardiovascular MRI (CMR) is an increasingly important tool in the investigation of cardiac disease 1. Like echocardiography, but in contrast with other imaging modalities such as computed tomography and positron emission tomography, CMR avoids exposing the patient to the risk of ionizing radiation which makes it a very attractive technique for both researchers and clinicians. However, perhaps the most significant challenge in the application of CMR techniques such as spectroscopy or perfusion imaging is that the signal-to-noise ratio (SNR) is often insufficient at established field strengths 2. As research in MR progresses, increasing static magnetic field strengths (B_0_) of MR scanners are becoming available. The use of ultra-high field (UHF) scanners, where B_0_ is greater than or equal to 7 Tesla (T), brings significant benefits. Notably, UHF MR scanners have been shown to provide increases in the achievable SNR and contrast-to-noise ratio (CNR) 3, which may extend the diagnostic capabilities of CMR.

The advantages of higher magnetic field strengths are counter-balanced by some significant complications which must be addressed. For instance the radiofrequency (RF) amplifiers used at 7T are very expensive; the peak power that can be generated is limited 4; and the heating in tissue from RF energy is greater at 7T than at 3T or 1.5T 5. A further major impediment to UHF imaging in the body is inhomogeneity in the RF transmit (B_1_^+^) fields 6,7. This occurs as a consequence of the increase in Larmor frequency with field strength, which results in a corresponding decrease in the RF wavelength. The RF wavelength at 7T (≈ 12 cm), is smaller than the dimensions of the human torso (approximately three–six wavelengths), so a pattern of constructive and destructive interference can be introduced to the B_1_^+^ field, resulting in regions of signal intensity and dropout in the images acquired. This inhomogeneity is compounded by attenuation of the RF energy due to the conductivity of the tissue 8.

Fortunately, there are techniques available by which B_1_^+^ inhomogeneity can be managed. For imaging of the brain, one method used to combat this problem is the use of high-dielectric pads 9 to increase the transmit field in an area local to the pad. Although a highly effective approach in the head at up to 7T, the depth of the heart in the chest cavity does not make this an attractive method, although some partial success has been seen in body imaging with this technique at 3T 10. Another recent method is the use of the Time-Interleaved Acquisition of Modes (TIAMO) scheme 11–13 whereby two time-interleaved images are acquired by exciting different B_1_^+^ modes and performing reconstruction by generalized autocalibrating partially parallel acquisitions (GRAPPA) 14 with the images representing two virtual receive elements. The different B_1_^+^ patterns are then used to calculate a more homogenous transmit field. In our work, we have used B_1_ shimming 5,15,16 which uses multiple RF transmit elements driven by independent amplifiers 6, allowing the phase and amplitude of these elements to be optimized to minimize image artifacts.

In a multi-element array, there are various coil designs which may be used. One such design is the transverse electromagnetic (TEM) resonator, which is an attractive option as it has shown good B_1_^+^ performance at UHF 5,17, it also shows decreased losses from radiation when compared with birdcage coils, improved current distribution and better quality factor under a load 18. However, each element in a TEM array must be individually tuned to the Larmor frequency and matched to the impedance of the transmit system to maximize the available B_1_^+^, which in this type of coil is sensitive to loading 18,19. Because these adjustments have historically been performed for every subject/phantom 2,20,21, much valuable scanner time is wasted. For instance, with an 8 channel cardiac array, this process takes approximately 30 min 22,23. In a research environment this is tedious but workable, however, in a clinical environment, a patient would be lying on the patient table for this time before any data being gathered, which is simply unacceptable.

In this work, we demonstrate a comprehensive system that remotely controls the tune and match of an RF transmit/receive cardiac array in a 7T MR scanner, by combining piezoelectrically operated tune and match capacitors, a dedicated control system, automated sensing of the tune state and a simple optimization algorithm. Furthermore, we demonstrate that it is possible to tune this array on multiple subjects at the magnet isocenter.

## METHODS

### Implementation

**Figure 1:**
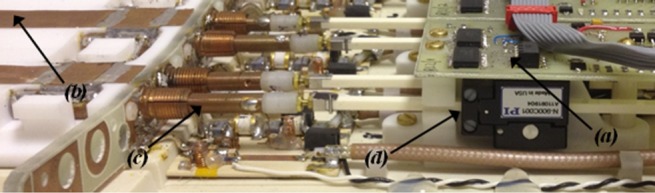
The inside of the posterior part of the RF array, showing (a) custom designed multiplexor/control boards, (b) the four coil elements, (c) the tune/match capacitor rods, and (d) the piezoelectric actuators.

The RF coil used was an eight-channel cardiac transceiver array consisting of posterior and anterior parts each with four microstrip TEM elements, 15.3 cm in length, 1.27 cm in width, and each spaced 5 cm from adjacent elements 17,20 with a low loss lower dielectric consisting of 2 cm of polytetrafluoroethylene (PTFE) and with air forming the upper dielectric. Each element has a dedicated pair of 1–10 pF variable rod capacitors 17 for adjusting the tune and match conditions 24. Originally, these capacitors were adjusted by manually manipulating a series of threaded tuning rods. For this study, the rods in the array were replaced by 16 piezoelectric stepping actuators (N310 NEXACT, Physik Instrumente, Germany). It has previously been shown that piezoelectric actuators may be used in an MR environment for the purpose of image-guided intervention and surgery 25,26. However, the actuators used in those studies were of the ultrasonic, hydraulic and pneumatic types. The system described here used a novel form of magnet safe linear actuator. The actuators are mounted on a runner 103 mm in length and contain piezoceramic elements which bend when charged to effectively “walk” along the length of the runner with an adjustable step-size of 5 nm to 5 µm. These actuators were fixed within the array housing and each connected to a tune or match capacitor, providing a force of up to 5 N. They were operated by means of a single controller unit (E-861 NEXACT, Physik Instrumente, Germany) with a custom built MATLAB (The Mathworks Inc., Natick, MA) software interface. Eight multiplexor/control boards were designed and built using optocouplers, allowing for the sequential control of each of the 16 actuators with the single controller. Each control board included limit detection, in the form of four optical switches (TCRT1000, Vishay Semiconductors, USA) to provide feedback to the controller when an actuator reached the end of its runner 22. This assembly can be seen mounted inside the array housing in Figure 1.

These additions to the array allow the capacitance to be altered remotely by command from a computer outside the magnet room (the “Auto-tune PC”). A custom MATLAB graphical user interface (GUI) and a Bluetooth enabled Wiimote controller (Nintendo, Japan) modified to be nonmagnetic, were both interfaced to the Auto-tune PC to control the actuators. This system made it possible to remotely tune and match the elements by manually controlling the actuators by means of the GUI or the Wiimote and monitoring the reflected power with an RF Sweeper box (Morris Instruments, Canada).

However, to further reduce the time required for tuning, a system was designed to entirely remove the need for manual calibration. Automated sensing of the tune state was achieved by connecting an RF phase and gain detector (AD8302, Analog Devices) to the forward and reflected directional coupler (DICO) ports of each of the eight RF amplifiers. The voltage output by the boards was then digitized by a 16-channel ADC (NI USB-6210, National Instruments, USA) connected to the Auto-tune PC. This voltage encodes a measurement of the ratio of forward/reflected RF voltage and relative phase (equivalent to the system scattering or “S-matrix”) across all eight channels. The S-matrix data gathered from the RF detectors was calibrated against a network analyzer (HP 8712C, Hewlett Packard, CA) at the head of the patient table to ensure accurate measurement. A constant offset in the measured S-parameters to account for cable losses was calculated based on the values given by the system for the standards of a shorted and an open circuit. This value was calculated to be +4.2 dB from the amplifier to the transmit panel at the head of the patient table where the coil is plugged in. The cables in the transmit chain are high performance, low loss Sucoflex 106 cables (Huber + Suhner, Switzerland). Thus, to within 13%, the magnitude |S_nn_| parameters according to the AD8302 datasheet equaled:

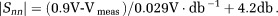


**Figure 2:**
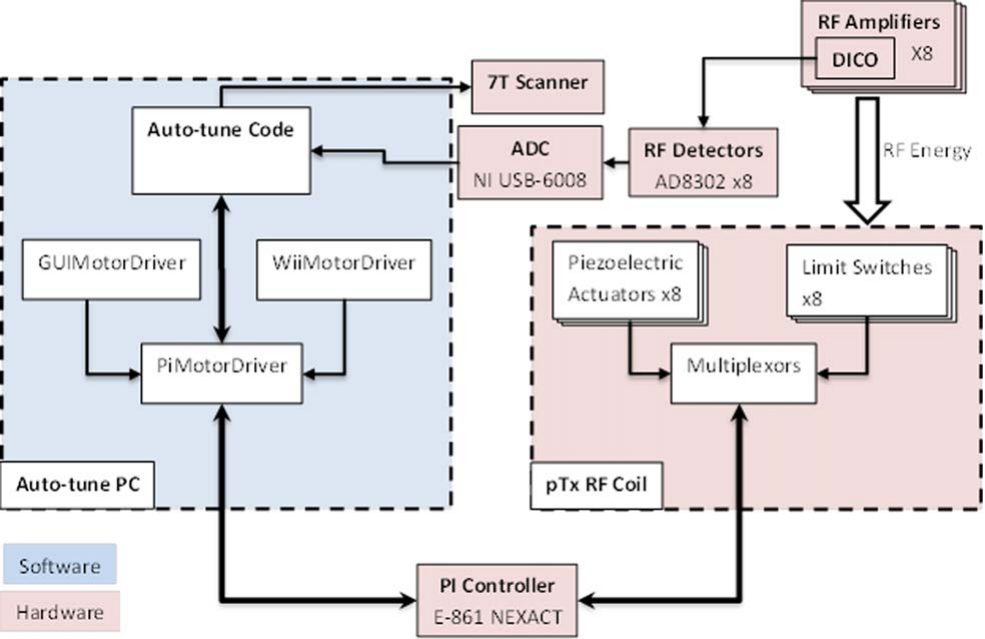
Data flow diagram of the whole auto-tune system, including the classes of the MotorDriver and the additional components of the RF array.

A pulse program was implemented on a Magnetom 7T scanner (Siemens, Germany) to pulse the different RF channels with 30 ms hard pulses at 30 V on the channel currently being tuned and 1 V on the seven remaining channels. This pulse program accepts commands over Ethernet from the Auto-tune PC. By measuring how much power is absorbed and reflected on each channel, it is possible to observe the effect of varying the tune/match capacitance. Hence, it is possible for the Auto-tune PC to perform a tuning operation. The full S-matrix (including all values of S_nm_) was acquired, allowing the effects of coupling between elements to be examined. For each element, the value which was optimized was |S_nn_|, which corresponded to the power response on the channel receiving the 30 V pulse, for instance |S_11_|, |S_22_|,… The data-flow of the entire system is described in Figure 2.

### Evaluation

To thoroughly characterize the performance of the actuators, the response of the elements and the effectiveness of the system, three sets of experiments were devised. In addition a simple auto-tune algorithm was devised and tested on a phantom and then for a total of nine runs performed on eight different subjects.

### Positioning Reproducibility

It was important to characterize how each of the actuators responded to the mechanical load of the capacitor rod, how multiple repetitions of the travel would affect the accuracy of the measured travel distance in actuator steps and whether the actuator performance was affected by the magnetic field. A MATLAB script was built to drive each of the piezoelectric actuators from one optical limit switch to the other, recording the number of steps required for each repetition. This was performed both on the bench and at the magnet isocenter. These data were used to assess how the forward and backward (push/pull) motion of the actuators compared, the effect of the magnetic field on the system and how much the measured distance varied between repetitions.

### Reflection Coefficient Profiles

The second stage of characterization was to describe the RF response of each coil element to ensure that the system is able to accurately and effectively determine the point of peak |S_nn_|. Hence, the S-matrix was measured over the entire two-dimensional (2D) (tune, match) search space. With a starting point of the actuator pair to be evaluated beginning at the negative limit and all other elements optimally tuned and matched, the relevant tune actuator was swept over its full range, recording S_nn_ every 100 steps (equating to approximately 1/30th of its full range). The match actuator was then moved by a single unit of 100 steps and the tune actuator again swept its full range. This was repeated until the match actuator had reached the opposite optical limit and required a mean of 21 min to complete. Plots were drawn of the |S_nn_| response of the element with a resolution of 100 actuator steps and the “acceptable region” of tune was marked. By convention this region was taken to be where the power loss from reflection was < 3%, or equivalently where |S_nn_| < −15 dB 27. This process was performed for each element on a volunteer and on a custom-made 21.7 liter saline drum phantom (70 mM NaCl + 3 mM gadobutrol, with conductivity to match that of muscle at 0.77 S/m).

### Performance Evaluation

The following simple auto-tune algorithm was implemented 23:

Optimize |S_nn_| with a sequential line search:

1. Element 1's match is stepped by +100 until a significant (> 5σ) increase in |S_nn_| is observed. *(a) If the first step reaches the end of the actuator's travel, or increases |S_nn_|, the direction is reversed*. (b) Element 1's tune is then optimized with the same process.
2. Step 1 is repeated until the position converges.
3. Step 1 is repeated for the remaining seven elements.
4. Steps 1–3 are then repeated until all elements move 100 steps or less in tune and match after an iteration and all |S_nn_| < −15 dB.

This algorithm was implemented in MATLAB.

To investigate the performance of the system as a whole, the array was loaded with the phantom, positioned at isocenter, and tuned automatically using the above algorithm before performing the following stress test. When the test began, it ran the auto-tune algorithm for three consecutive iterations. An offset was then applied to each channel to move the tune away from the optimal value before the algorithm was run again for another three iterations. The value of this offset was randomized over the eight actuators, by a starting value which increased in units of 400 (0, 400, 800, … , 4000, where 4000 is the maximum range achieved by any of the actuators) being multiplied by a matrix of eight random numbers ranging from −1 to 1. For every iteration of the process, the S-matrix was recorded, as was the elapsed time. Following the third and final iteration for each value of the offset the actuators were each driven to the negative limit. The steps required for this move were counted such that absolute final positions for the tune solution could be recorded and compared.

### Volunteer Tests

Eight volunteers were recruited in accordance with the ethical practices of our institution. A total of nine tests of the autotuning system were subsequently run on the eight different volunteers. Each volunteer lay supine on the posterior array with the anterior array in place on top of their chest. All volunteer tuning experiments took place at the magnet isocenter. The auto-tune algorithm was run for three consecutive iterations on each subject (a total of 27 iterations). Final |S_nn_|, final actuator positions and the time elapsed were all recorded. For each test, the actuators began in a position close to the middle of their range of travel. For subsequent runs, the starting point was the final position from the preceding iteration.

### Coupling

Some further work was done to investigate the effects of coupling between transmit elements 23 (represented by the S_nm_ terms of the gathered S-matrix). An additional optimization algorithm was implemented which optimized the total reflected power Σ_m_S_nm_, instead of optimizing |S_nn_| for each channel individually. It was found that the coupling between elements (particularly central elements: 1/2 and 6/7) led to the tune solution of channels adjacent to that being tuned becoming marginally worse. However, using this method proves to be challenging because the minima are shallow and hard to fit and any improvement in tune solution is modest. Using this approach also makes it somewhat subjective what the optimal solution actually is and typically S_nm_ is above −6 dB in this well designed coil.

### Imaging and B_1_ Maps

A further experiment was devised to show the effect on image data of using a tailored tune solution for every subject. Autotuning was performed on four volunteers and the final actuator positions saved in the GUI. The array was then tuned and matched on a volunteer, and a FLASH cine sequence (TE = 3.3 ms, TR = 71.1 ms, for a flip angle of 35° and resolution of 1.4 × 1.4 mm^2^) was acquired in the mid-ventricular short axis of the left ventricle (LV) of the heart. The actuators were then driven to a saved position representing the tune solution from one of the previous subjects and the imaging sequence was repeated. This was done for each of the saved solutions. The array was then re-tuned to the volunteer in the magnet and the cine repeated once more.

Analysis of all the images collected was performed in MATLAB. Regions of interest (ROIs) were drawn on the images around the myocardium and divided into the standard segments 28. The difference in the signal intensity in images acquired with different tune solutions could then be analyzed.

## RESULTS

### Positioning Reproducibility

Results were gathered for reproducibility experiments of 100 repetitions performed both on the bench and at field. Separate values were recorded for both tune and match on each channel. The mean number of steps and the standard deviation (SD) for each direction were calculated, along with the difference in steps in each case when at B_0_ compared with no field. Note that a step is defined as a single movement of the piezoelectric bending elements of the actuator. Each actuator takes between 2500 and 4000 steps to cover the range of motion of its associated tune/match capacitor.

Comparing the number of steps forward and backward, the largest difference was for the actuator controlling the match capacitor of channel 5 (5M), which took 3269 ± 65 steps forward (mean ± SD) and 2805 ± 11 steps backward on the bench, i.e., a difference of −17%. The lowest difference was 2% for channel 7M. The SD on channel 5M was 2% forward and just 0.4% backward. The SD was always < 5% of the full range of motion.

Furthermore, comparing results at isocenter and on the bench, there is a difference of between +8% and +19% in the number of steps at isocenter compared with on the bench. For instance, channel 5M took mean values of 3545 ± 42 and 3205 ± 15 steps in the forward and backward directions, respectively, compared with the values presented above.

### Reflection Coefficient Profiles

**Figure 3:**
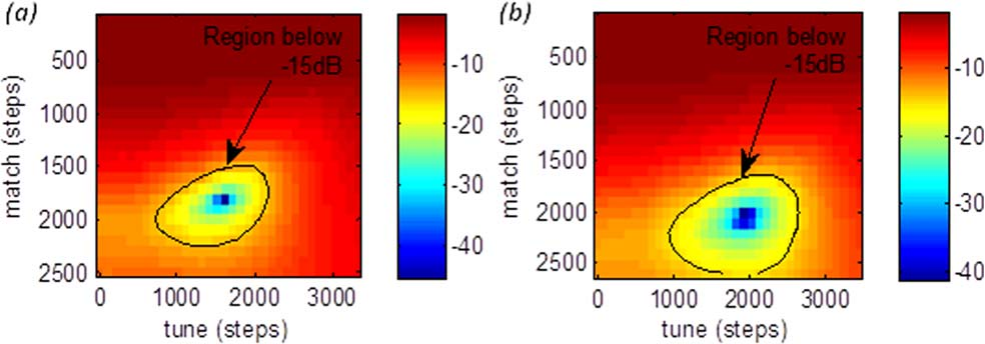
Tune/match profiles for a single element of the RF array showing the |S_nn_| profile for (a) a large saline drum phantom, and (b) for a volunteer. The “acceptable region” of tune (|S_nn_| < −15dB) is marked.

Tune/match profiles were gathered for each coil element using the large saline phantom and a human volunteer. Representative data for a single element is shown in Figure 3 for the phantom (Fig. 3a) and in vivo (Fig. 3b). These profiles give the |S_nn_| response across all of 2D tune/match space. These profiles show clear peaks in the power that is delivered to the load. Around the point of minimum |S_nn_| (1600, 1800, −45.42 dB) for the phantom, there is a substantial region, marked in Figure 3a, where the power ratio is less than −15 dB (“acceptable tune”). For the volunteer, the results were similar, except that there is a shift in the acceptable region and the point of minimum |S_nn_| (1900, 2000, −41.25 dB).

### Performance Evaluation

**Figure 4:**
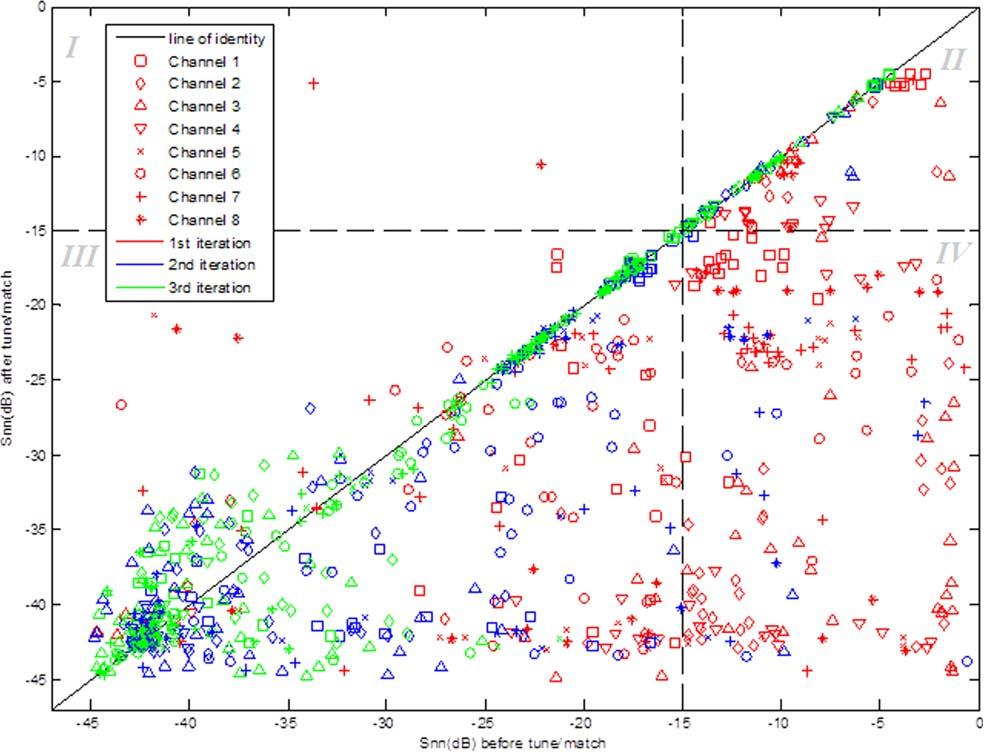
Results of the evaluation of auto-tune algorithm performance on a phantom. The value of the S-parameter before the algorithm was run is plotted against that recorded after the tuning process. Points shown in red are data for the first iteration of the experiment, while the blue and green points are data for the second and third iterations, respectively. The concentration of points on or below the line of identity shows the positive effect of the tuning algorithm on the S-matrix. The plot is divided into four quadrants I–IV at the −15 dB cut-off in both dimensions.

The simple auto-tune algorithm used in this work achieved |S_nn_| < −15 dB in 85.7% of iterations of the algorithm. In Figure 4, all the points in quadrants III and IV have S-values < −15 dB after tuning and represent a successful outcome. The data in quadrant III lies below the cut-off before and after tuning and the points in quadrant IV are those which are improved from above −15 dB to below by the tuning process. The two points in quadrant I were made worse by the algorithm. The points in quadrant II have |S_nn_| > −15 dB both before and after tuning. Data points below the line of identity represent values that were improved by the algorithm.

### Volunteer Tests

On six of eight of the volunteers, the algorithm was able to find an acceptable tuning solution (i.e., |S_nn_| were all < −15 dB) on the first run; the following two runs provided fine tuning. On the other two volunteers, the algorithm failed initially to find a tuning solution for just one of the eight elements but further runs proved successful. In every test, the first cycle of optimization completed in a mean of 5 min 10 s ± 38 s; and the second and third runs, where the starting position was the final position following the previous run, each completed in 2 min or less.

### Imaging and B_1_ Maps

**Figure 5:**
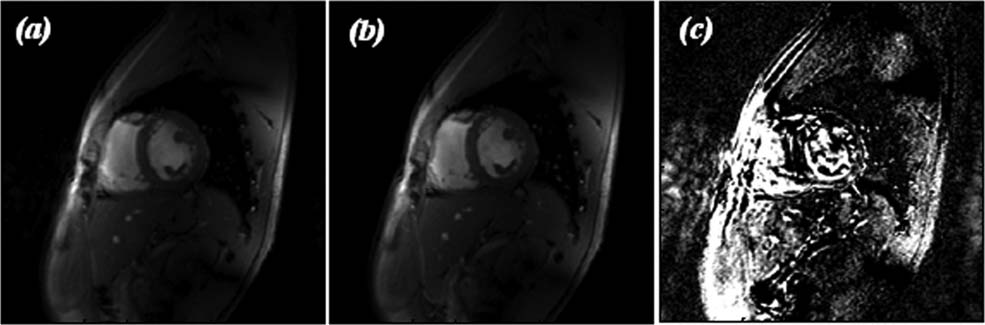
Example of FLASH cine images for (a) the auto-tune solution for the volunteer, (b) a saved solution for a previous volunteer, and (c) a subtraction of the 2 images to show the difference in pixel intensity.

Figure 5 shows a frame from the FLASH cine from the same point in the cardiac cycle in three forms. The image in Figure 5a was acquired with the tune solution for the volunteer. Figure 5b is the equivalent frame from the cine taken after the solution has been changed to that acquired for one of the previous volunteers. A subtraction was then performed on these two images to show the difference in pixel intensity between the two solutions. This is presented as Figure 5c.

The difference in intensity relative to the auto-tuned data was calculated for the images collected using previous subjects' solutions. These values range from 3% to 22% across the six segments but was typically between 7 and 15%.

## DISCUSSION

### Positioning Reproducibility

Each of the actuators was observed to require an appreciably different number of steps when travelling to the negative limit (pull motion) than when travelling to the positive limit (push motion). However, the ratio between forward and reverse steps remained consistent for each actuator. In percentage terms this difference was in the range of 2 to 17%. We believe that this is due to the difference in force required to push or pull the capacitor rod. In future, we will eliminate this complication by optimizing in units of “forward motor steps”, applying a constant calibration factor for the reverse motion of each actuator.

The number of actuator steps required to cover the full range of motion depended on the presence of a magnetic field. We compared this response using the same coil loading and cable length on the patient table such that only B_0_ changed. It was shown that the actuators consistently required around 15% more steps at full field than on the bench. The switching threshold for the optical limit switches was also affected by the magnetic field. Both of these differences were found to be caused by the same phenomenon, whereby the amount of current that flowed to the limit switches was affected by B_0_. Thus, the value of the voltage required to change state was different, but also the point in space at which the switch detects the presence of the actuator shaft and is “switched” was later in its travel, leading to an increased actuator range when at field. This was not perceived to be problematic in this implementation, as one of the advantages of the system presented here is that tuning can always take place at the isocenter of the magnet. However, it is something to be aware of when designing systems of this sort.

### Reflection Coefficient Profiles

The S_nn_ profiles in Figure 3, along with those collected for the other elements, characterize the power response of each element. As described previously 18,19, TEM coil elements are more sensitive to coil tuning than other coil designs, as the B_1_ field is more sensitive to loading from a sample or subject. Figure 3 demonstrates that there is a significant gradient of |S_nn_| across 2D tune/match space. These profiles also show clearly that our S-matrix measurement apparatus is sensitive enough (±0.02 dB) to allow an automated algorithm to find the region of minimal reflected power.

In Figure 3, the “acceptable region” comprises some 1000 × 1200 actuator steps for the phantom (Fig. 3a), and a similar region of 1100 × 1100 steps in vivo (Fig. 3b). The other channels show similarly sized “acceptable regions.” The algorithm moved in units of 100 steps, a number chosen to be small compared with the typical size of the “acceptable region.” This affords a compromise between ensuring adequate precision in the final actuator positions and ensuring a speedy completion of the tuning process.

Figure 3 also shows that there is a shift in the position of the “acceptable region” with different coil loading, i.e., comparing results for the phantom and the different volunteers. The peak point of |S_nn_| differs by 300 steps in the tune direction and 200 steps in the match direction for this element between the phantom and the volunteer. If the array were tuned on the phantom and the point (1400, 1600) in tune and match were chosen, this would give a perfectly acceptable |S_nn_| value of −15.65 dB. However, this same solution achieves only −10.02 dB in the volunteer. Operating at 7T with 8 × 1 kW amplifiers we currently are somewhat limited in the peak B_1_ that can be achieved. It is, therefore, important to minimize the power that is lost through reflection. This system also provides a tool with which to quantitatively explore the S matrix in the 2D tune/match space, allowing us to explore the impact of tuning on every subject; whether to store preset solutions for different body-shapes/genders etc.; or simply run the process periodically as part of a quality assurance routine. To date the variation in subjects has been small (n = 8; age = 33 ± 12 years; BMI = 24.2 ± 3.4 kg/m^2^, all male) and so by not tuning per subject the lost B_1_^+^ was fairly minimal. However, we see substantial tune and consequently B_1_^+^ variation between subjects and phantoms and expect effects of similar magnitude once we image typical cardiac patients with high BMI.

### Performance Evaluation

Figure 4 shows that the final optimized tune and match met the −15 dB criterion for “acceptable tune” in all coil elements 85.7% of the time over 4 runs. For the remaining 14.3%, it should be noted that this performance evaluation was designed to test the auto-tuning algorithm extremely rigorously. It used some starting positions that were very far from the known average region of tune and with potential extremes of positioning (such as paired tune/match actuators starting close to opposite limits), yet this simple algorithm could still find an acceptable solution in 85.7% of cases. In experimental or clinical use these extremes would not occur, therefore, in practical applications, the accuracy of the algorithm is expected to be considerably higher. The concentration of points below the line of identity shows the positive effect of the algorithm on the S-matrix in the majority of cases. Finally, the green points, denoting the third iteration of auto-tuning, are concentrated around the line of identity, which shows that the first two iterations contribute most, with the third often having little further effect. Therefore, time could be further reduced by only performing two iterations of the process.

Data was also gathered over the course of the experiment to show how the time to find a tune solution varies. This data suggests that the first pass performs the gross actuator movements to give a coarse tune solution which is refined in the second and third iterations.

### Volunteer Tests

In the 27 volunteer tests (3 iterations on 9 occasions) with eight subjects, the auto-tune algorithm successfully found an acceptable solution in all but two cases. However, in these two cases the resulting |S_nn_| was only marginally above −15 dB and was improved by further iterations.

The quicker completion and better |S_nn_| from the second and third runs on the successful subjects shows that the algorithm would be even more efficient if a better starting position were chosen (e.g., a previous solution for the particular subject or an average starting point for new human subjects). This would be equivalent to the second and third runs from this experiment, so the whole tuning process would be expected to require approximately 2 min per subject. To this end, a feature of the MATLAB interface was created which allows the user to save the actuator positions and return to them later.

This auto-tune system also allows for the array to be more easily tuned and matched at isocenter to compensate for the effects of the magnetic field, patient table or the bore liner on the coil loading or decoupling. The system as a whole is much less demanding to use than the original setup and so this, along with the reduced time and improved subject experience, should facilitate the introduction of clinical studies in the body on a 7T scanner using a multiple element transceiver array.

### Imaging and B_1_ Maps

As mentioned previously, due to the limited peak B_1_ power available at 7T, it is desirable to ensure that all the available power is delivered efficiently to the subject. Figure 5c demonstrates that minimizing the power lost through reflection by auto-tuning on individual subjects can lead to an improvement in image quality, as there is more signal available when a tailored tune solution is used.

## CONCLUSIONS

We have shown that it is possible to use piezoelectric actuators for tuning and matching an eight-channel TEM cardiac RF array in a 7T MR scanner. We have demonstrated the use of forward vs. reflected power values recorded at the RF amplifiers to evaluate the tune/match, and that this process can be automated. Auto-tuning on a human subject can be performed routinely in less than 10 min, compared with a previously reported mean of 30 min for manual tuning 22,23 making 7T cardiac MRI more feasible on patients when the coil systems used require tuning. Furthermore, this system has now entered into routine prescan use in our lab for both volunteers and clinical studies.
